# A Protein Required for Fruitflies to Dispatch Wasp Parasites

**DOI:** 10.1371/journal.pbio.0020255

**Published:** 2004-08-17

For a three-millimeter invertebrate, the Drosophila fruitfly has a remarkably sophisticated immune system. Granted, it can't customize an immune response by grooming cells to “remember” and target specific pathogens. But it can rally the less specialized tools of innate immunity to fight disease and infection, and in so doing draws on several aspects of blood cell development (called hematopoiesis)—the foundation of the cellular immune response—also found in vertebrates.

In fruitflies, as in vertebrates, hematopoiesis occurs in distinct stages and locations, with nascent cell populations migrating to establish new hematopoietic frontiers. A population of progenitor cells generates all the blood cell types in the organism. These cells arise in two distinct waves and in two distinct locations, with their progeny differentiating into the specialized tissues and organs of the hematopoietic system. In mammals, these organs include the liver and bone marrow, an ongoing source of blood cells after embryogenesis. In fruitflies, the definitive hematopoietic organ is the lymph gland, which churns out three types of blood cells: plasmatocytes and crystal cells, which are also produced by embryonic hematopoietic precursors, and lamellocytes. Plasmatocytes account for up to 95% of circulating fruitfly blood cells and act much like their mammalian counterpart, the macrophage, engulfing substances deemed foreign and dangerous. Crystal cells account for most of the rest and are involved in melanization reactions, which trigger mechanisms involved in containing and killing invading microbes. Unlike plasmatocytes and crystal cells, lamellocytes appear en masse and only under certain conditions, such as the unwelcome appearance of parasitic wasp eggs. Lamellocytes encapsulate and neutralize the invader.[Fig pbio-0020255-g001]

**Figure pbio-0020255-g001:**
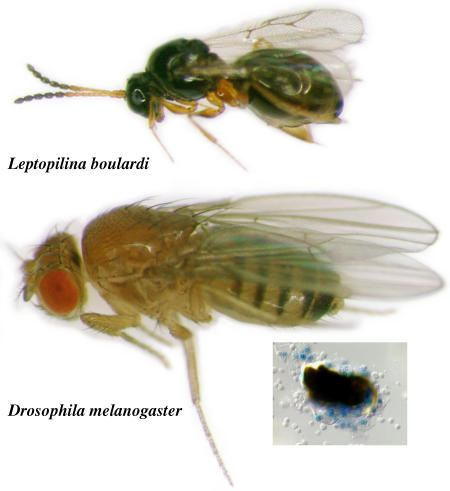
Cellular immune response to parasitization in Drosophila requires the EBF ortholog Collier

Molecular factors involved in determining the fate of hematopoietic cells have been identified for plasmatocytes and crystal cells but not for lamellocytes—until now. In a search for genes that might precipitate lamellocyte differentiation, Marie Meister, Alain Vincent, and colleagues homed in on a protein, called Collier (Col), that is expressed in lymph glands at the end of embryogenesis. Col is quite similar to a mammalian protein, called Early B-cell Factor (EBF), that controls B-cell development in mice. Both proteins are transcription factors, exerting control by initiating gene transcription.

To investigate Col's part in lamellocyte development, the researchers had to get around the fact that mutations that render Col nonfunctional eventually kill the embryo. Using tricks of the genetics trade, Meister and colleagues generated fly larvae that survive loss-of-function mutations in the gene that encodes Col, allowing them to study its role in hematopoiesis.

The mutants had normal amounts of circulating plasmatocytes and crystal cells, but when exposed to parasitic wasp eggs, could not muster the requisite response: lamellocyte production. With no lamellocytes, fly larvae had no means of protection against encroaching wasp eggs, which, uncontested, developed into parasitic larvae. The flies with normal Col levels had no such problem, producing considerable numbers of lamellocytes. In these flies, Col expression was restricted to a lymph region called the posterior signaling center (PSC). Col's influence on lamellocyte fate was strong enough that forcing Col expression in precursor blood cells induced lamellocyte differentiation even in the absence of wasp infestation. Based on these findings, Meister and colleagues propose a model for Col-mediated lamellocyte differentiation in which wasp infestation activates Col-expressing cells in the PSC, which then instructs immature blood cells in the lymph gland to become lamellocytes and dispatch the gathering threat.

Col's role in fruitfly hematopoiesis closely parallels that of its mammalian ortholog in white blood cell development, EBF. Both are required to generate specialized populations of cells in response to a particular immune threat, and both confer an extra line of defense when faced with special circumstances—key features of vertebrate adaptive immunity. Could it be that building blocks of adaptive immunity were already in place some 550 million years ago, when flies and vertebrates parted ways? Researchers will have to investigate the molecular agents of immunity in intervening species to find out.

